# Prevalence and risk factors for depression in women with multiple sclerosis: a study from Iran

**DOI:** 10.1186/s12991-015-0069-8

**Published:** 2015-09-22

**Authors:** Khadijeh Mohammadi, Parvin Rahnama, Ali Montazeri

**Affiliations:** Department of Midwifery, Faculty of Nursing and Midwifery, Shahed University, Tehran, Iran; Mental Health Research Group, Health Metrics Research Centre, Iranian Institute for Health Sciences Research, ACECR, Tehran, Iran; Faculty of Humanity Sciences, University of Science and Culture, ACECR, Tehran, Iran

## Abstract

**Background:**

Multiple sclerosis is increasingly becoming a major health problem among women worldwide. The aim of the present study was to estimate prevalence of depression in women with multiple sclerosis and also to identify risk factors contributing to its development.

**Methods:**

This was a cross-sectional study of depression in a sample of 226 women with multiple sclerosis. The sample was recruited from an outpatient clinic in Tehran, Iran. Depression was assessed using the Beck Depression Inventory-II (BDI-II). Univariate and multiple logistic regression analyses were performed to examine the association between depression and independent variables.

**Results:**

Overall, 91 women (40.2 %) had moderate to severe depression. The mean age of participants was 35.7 years (SD = 8.07). The results obtained from multiple logistic regression analysis showed that the disease course (OR for relapsing–remitting MS = 2.36, % 95 CI = 1.14–5.53, *P* = 0.46), the expanded disability status scale (OR for score of 5–8 = 4.88, % 95 CI = 2.51–11.06, *P* < 0.001) and employment status (OR for housewife = 4.75, % 95 CI = 1.55–14.58, *P* = 0.006) were significant contributing factors to depression in patients with multiple sclerosis.

**Conclusions:**

The findings suggest that depression in patients with multiple sclerosis is multi-factorial and very much dependent to physical and social conditions of patients. The recognition of such conditions might help clinicians to manage patients more effectively.

## Background

Multiple sclerosis (MS) is a chronic and functional disabling disease, occurring from demyelination of the CNS nerve fibers [1]. During the last decade there was dramatic increase in the incidence and prevalence of MS in Iranian population and it was reported that female/male ratio ranged from 1.8 to 3.6 [2, 3]. Similarly a western study found that the real increased incidence was limited to women [4]. A recent study suggested that the prevalence of MS in Iran is even higher than neighboring countries and within Iran itself the prevalence varies by geographical areas. One can speculate that genetic and environmental factors might have contributed to these observations [3]. It is well documented that one of the environmental risk factors for developing MS is vitamin D insufficiency [5]. On the other hand, there is increasing evidences for the vitamin D deficiency among Iranian women [6–8].

The disease has several adverse effects on patients’ life. Patients with multiple sclerosis not only suffer from physical disability but also experience psychological distress more often. Depressive disorders are more prevalent in MS patients compared to other chronic neurologic disorders [9]. Thus, depression is often underreported and under-diagnosed in patients with multiple sclerosis.

In a study from Portugal, Silva et al. found that there were significant associations between depression and several factors including age, disease duration, age at onset, and functional impairment in MS patients [10].

Chwastiak and Ehde summarized the literature and suggested that depression in MS patients may contribute to cognitive dysfunction, decreased quality of life, unemployment, and decreased adherent to treatment regimens for MS compared to non-depressed MS patients [11]. Furthermore, it was shown that MS patients with depression develop fatigue about four times more than non-depressed MS patients [12]. It remains unclear whether a secondary response to the illness is the reason of depression or the neurobiological effects are the origins of depression [10].

It is crucial for the health care providers to have a better understanding about the identification of patients who are at risk of developing depression in this community. However, if risk factors for depression identified, it may lead to the timely initiation of appropriate treatment and to produce efficient strategies to lowering MS burden as soon as possible. To the best of our knowledge, there are a few studies about depression in MS patients in Iran. A study of 106 MS patients indicated that physical disability and depression strongly influenced quality of life in these patients [13]. Another investigation from Iran showed that depression in MS patients might cause fatigue and sleep disturbances [14]. However, none of these studies indicated risk factors for depression in MS patients. The aim of the present study was to estimate prevalence of depression in Iranian women with MS and also to identify risk factors that contribute to its development.

## Methods

### Design and procedure

This was a cross-sectional study that carried out to assess depression in female patients with multiple sclerosis. A detailed design is explained elsewhere [15] but in summary a total of two hundred and twenty-six female patients with multiple sclerosis were recruited consecutively from an MS outpatient clinic in Tehran, Iran. Criteria for inclusion were: diagnosis of MS according to the McDonald Revised criteria [16], be married; having the Expanded Disability Status Scale (EDSS) score ≤ 8 [17] and willingness to participate in the study. It is worth noting that the current study was as part of an investigation on sexual dysfunction in women with multiple sclerosis. Since for Muslims sexual function out of marriage is forbidden we only studied married women. Women with pre-existing major chronic illnesses such as depression were excluded. All patients had a neurologic examination.

### Measures

1. Sociodemographic and clinical characteristic included recording of age, education, employment status, age at onset of disease, disease duration, disease course, and degree of disability. This latter information was collected by using the Kutzke Expanded Disability Status Scale (EDSS). In fact, the EDSS assesses the neurological impairment in women with MS patients [17]. The score on the EDSS ranges from 1 to 10. Scores from 1.0 to 4.5 specify that patients are fully ambulatory while scores from 5.0 to 9.5 indicate that patients are severely impaired and death defines a score of 10.
2. Depression: The Beck Depression Inventory-II (BDI-II) was used to assess the presence and intensity of depressive symptoms [18, 19]. This instrument consists of 21 items. Each item is rated on a 4-point Likert scale ranging from 0 to 3 and thus giving a total score of 0–63, with higher scores indicating more frequent depression symptoms. A cutoff point of 19 and higher was considered as moderate to severe depression [20]. Psychometric properties of the Iranian version of the BDI-II are well documented [21].

### Analysis

Descriptive statistics was used to explore the data. Participants were classified as with and without depression based on the Beck Depression Inventory-II (BDI-II). Univariate analysis was performed to indicate the association between dependent (depression) and independent variables. Then, to determine the risk factors for depression in MS patients, multiple logistic regression analysis was carried out while controlling for all independent variables. The reference level for age was age < 35 years old versus aged 35 years old and older; for education was higher level versus primary/secondary level; for employment status was employed versus not employed; for disease duration was 0–8 years versus ≥ 9 years; for disease course was RRMS versus PPMS/SPMS; for EDSS was score 0–4.5 versus 5–8; and for depression treatment was yes versus no. The SPSS version 16 was used to analyze the data in two steps.

### Ethics

The ethics committee of Shahed University approved the study. We obtained written informed consent from participants after comprehensive explanation of procedure involved.

## Results

### Sociodemographic and clinical characteristics of the study sample

In all 226 women with multiple sclerosis were studied. The mean age of participants was 35.7 years (SD = 8.07). The mean disease duration and age at onset of the disease were 1.84 (SD = 0.79) and 29.51(SD = 7.65) years, respectively. The mean depression severity score (BDI) was 17.86 (SD = 11.13). The disease course was as follows: 169 of patients (74.8 %) had relapsing–remitting MS (RRMS), 4 (1.8 %) had primary progressive MS (PPMS) and 53 (23.5 %) had secondary progressive MS (SPMS). There were 165 patients (73.0 %) with the EDSS score less than 4.5. One hundred and fifty-seven patients (69.5 %) had low educational levels and 189 patients (83.6 %) were housewife. Overall, 91 women (40.2 %) had moderate to severe depression. The detailed results are shown in Table 1.

**Table 1:** Characteristics of the study sample

	With depression (*n* = 91)	Without depression (*n* = 125)	OR (95 % CI)^a^	*P*
	No. (%)	No. (%)		
Age (years)				
≤35	45 (49.5)	72 (53.3)	1.0 (ref.)	
>35	46 (50.5)	63 (46.7)	1.16 (0.68–1.98)	0.567
Education				
Higher	21 (23.1)	48 (35.6)	1.0 (ref.)	
Primary/secondary	70 (76.9)	87 (64.4)	1.83 (1.00-3.35)	0.046
Employment status				
Employed	5 (5.5)	103 (76.3)	1.0 (ref.)	
House wife	86 (94.5)	32 (23.7)	5.34 (1.99–14.31)	<0.001
Age at onset of disease (mean/SD)	28.75 (7.71)	30.02 (7.60)	0.97 (0.94–1.01)	0.225
Disease duration (years)				
0–8	62 (68.1)	107 (79.3)	1.0 (ref.)	
≥ 9	29 (31.9)	28 (20.7)	1.78 (0.97-3.27)	0.050
Disease course				
RRMS	66 (72.5)	103 (76.3)	1.0 (ref.)	
PPMS and SPMS	25 (27.5)	32 (23.7)	0.82 (0.44–1.50)	0.522
EDSS score				
0–4.5	52 (57.1)	113 (83.7)	1.0 (ref.)	
5–8	39 (42.9)	22 (16.2)	3.85 (2.07–7.14)	< 0.001
Received treatment for depression				
Yes	7 (7.7)	15^b^ (11.1)	1.0 (ref.)	
No	84 (92.3)	120 (88.9)	0.66 (0.26–1.70)	0.398

^a^Derived from univariate logistic regression analysis

^b^In fact, these patients were indicated as ‘without depression’ based on cutoff value of the Beck Depression Inventory; otherwise clinicians recognized these patients as having depression and thus received treatment

The EDSS score for RRMS and PPMS/SPMS is shown in Table 2. As shown, most patients with RRMS scored 0–4.5 while a small number for patients with PPMS/SPMS scored 0–4.5 (86 vs. 35 %).

**Table 2:** EDSS scores of RRMS versus PPMS and SPMS groups

	RRMS	PPMS/SPMS
	No. (%)	No. (%)
EDSS score		
0–4.5	145 (86)	20 (35)
5–8	24 (14.4)	37 (65)
Mean (SD)*	2.20 (0.16)	2.08 (0.27)

* *P* value for mean difference = 0.095

### Risk factors for depression

The association between depression and independent variables was first examined by univariate analysis. The results showed that there were significant associations between depression and the disease duration (*P* = 0.050), the EDSS (*P* < 0.001), educational level (*P* = 0.046) and employment status (*P* < 0.001). However, no significant associations observed between depression and age (*P* = 0.567), the disease course (*P* = 0.522), age at onset of the disease (*P* = 0.225) and depression treatment (*P* = 0.713). The results are shown in Table 1.

As shown in Table 3 the results obtained from multiple logistic regression analysis indicated that the disease course (OR for relapsing–remitting MS = 2.36, % 95 CI = 1.14–5.53, *P* = 0.46), EDSS (OR for score of 5–8 = 4.88, % 95 CI = 2.51–11.06, *P* < 0.001) and employment status (OR for housewife = 4.75, % 95 CI = 1.55–14.58, *P* = 0.006) were significant contributing factors to depression in patients with multiple sclerosis.

**Table 3:** The results obtained from multiple logistic regression analysis indicating risk factors for depression (*n* = 226)

	OR (95 % CI)	*P*
Age (years)		
>35	1.0 (ref.)	
≤35	1.03 (0.54–1.97)	0.908
Education		
Higher	1.0 (ref.)	
Primary/secondary	1.09 (0.51–2.3)	0.815
Employment status		
Employed	1.0 (ref.)	
House wife	4.75 (1.55–14.58)	0.006
Disease duration (years)		
0–8	1.0 (ref.)	
≥ 9	1.34 (0.65–2.76)	0.420
Disease course		
RRMS	1.0 (ref.)	
PPMS and SPMS	0.42 (0.18–0.98)	0.046
EDSS score		
0–4.5	1.0 (ref.)	
5–8	4.88 (2.15–11.06)	< 0.001
Age at onset of the disease	0.96 (0.93–1.00)	0.115
Depression treatment		
Yes	1.0 (ref.)	
No	1.21 (0.43–3.33)	0.713
2 log-likelihood test	267.24	
Nagelkerke R2	0.206	
Global percentage of identified cases	69.5	

## Discussion

The findings from current study showed that the prevalence of depression among Iranian female with MS was relatively high. As suggested this high prevalence might be explained by various reasons including structural brain damage [22], physical disability [23], treatment medication [24], and severity of MS. Unfortunately, it was reported that two-thirds of depressed MS patients receive no treatment [25].

Physical disability considered as common MS consequence that may be associated with depression in this community. However, the relationship between physical disability and depression in MS patients remains controversial [26]. Perhaps assessing link between depression and physical disability in MS patients needs a cohort design. However, unlike the results of studies that did not find any association between physical disability and depression in MS patients [27, 28] the current study showed that functional impairment was a significant contributing factor to the outcome. It seems that the depression worsens as the disease progresses. On the other hand, consistent with our study, Chwastiak et al. reported that MS patients with advanced disability were six times more likely to experience depressive symptoms than those reporting minimal severity [29]. One possible explanation is that the physical problems might be impeding participation in recreational activities and also it was associated with occupational problems [30]. The result of study revealed that disability in patients with multiple sclerosis is age dependent. It is argued that the disability milestone was reached at about the same age in different courses of disease [31]. It might be explained by the fact that in this study the patients with RRMS were older than PPMS/SPMS.

The effect of unemployment has not been suitably investigated in MS patients. This study showed that 189 women (83.6 %) with MS were unemployed. This high unemployment status in MS patients might be explained by different variables including neurological, cognitive status, anxiety and depression [32]. Overall women’s unemployment rate is 20.9 % according to the latest report released by the Statistical Center of Iran [33].

We found that women with RRMS experienced higher rate of depression compared with women at progressive stage. Similarly, Zabad et al. found that patients with primary progressive MS had a lower lifetime risk of major depression compared with patients with relapsing forms of the disease. It was suggested that perhaps the PPMS group might have received more adequate social support, which may have conferred a protective effect for depression occurrence in these patients. In addition, increased inflammatory components were reported in the relapsing forms of MS that may be associated with depression [34]. However, the association between the course of disease and depression was found to be inconsistent when relapsing–remitting MS was compared with progressive MS [35]. Other investigators found that depression symptoms were persistent even during remission from MS [36, 37]. There is evidence that 85 % of MS patients who were diagnosed with RR experienced a stressful life event. Accordingly, it could be argued that a higher rate of anxiety and depression among this group of patients might be associated with worse adjustment problems [38, 39].

## Limitations

The findings from this study should be interpreted with caution since the study had some limitations. This was a cross-sectional study while longitudinal studies are needed to establish link between depression and multiple sclerosis. It is argued that depression in this patient might be due to several reasons including the fact that it may precede the onset of the disorder (being a risk factor for actions on immune system), may be a reaction to the disease (principally, as adjustment disorder), may be a consequence of the therapy (interferon, for example) and may depend on brain lesions caused by the disease.

## Conclusion

The findings indicated that depression was frequent in women with multiple sclerosis. Identifying factors associated to depression in MS patients is important because this may allow clinicians to address some potentially reversible causes or manage patients more efficiently at earlier time.
